# Editorial: Livestock Systems in Urbanizing Environments: Impacts and Implications for Food Security in Developing Countries

**DOI:** 10.3389/fvets.2022.966044

**Published:** 2022-06-30

**Authors:** Assem Abu Hatab, Sofia Boqvist, Carl-Johan Lagerkvist

**Affiliations:** ^1^Department of Economics, Swedish University of Agricultural Sciences, Uppsala, Sweden; ^2^Nordic Africa Institute, Uppsala, Sweden; ^3^Department of Biomedical Sciences and Veterinary Public Health, Faculty of Veterinary Medicine and Animal Science, Swedish University of Agricultural Sciences, Uppsala, Sweden

**Keywords:** livestock systems, animal-production, urbanization, food-security, low- and middle-income countries (LMICs)

In recent decades, rapid urbanization in low- and middle-income countries (LMICs) together with other demographic, socioeconomic, and environmental changes have brought urban livestock keeping back on the urban development agenda (Lindahl et al.). On the one hand, the increased attention both by research and policy to urban livestock keeping in LMICs' cities is attributable to the well-documented contributions that livestock systems make to livelihoods, poverty reduction, food security, and wellbeing of the poor and under-nourished groups (1). On the other, urbanization in most of LMICs has been associated with high rates of urban sprawl that is substantially changing the landscape of livestock production, increasing the complexity of livestock supply chains, and putting pressures on the capacity of livestock systems to meet the growing demand for animal-source food (1). Therefore, there have been increasing uncertainties about how urban livestock systems could adapt to meet the profound challenges that many LMICs' cities are projected to face in relation to hunger, food insecurity and malnutrition. This has also amplified the need to accelerate efforts to build more sustainable and resilient urban livestock systems that foster food security to meet the needs of the increasing population pressure and enable LIMCs to achieve the sustainable development goals (2).

This Research Topic was put forward with the purpose to provide greater understanding of urban livestock systems in various LMICs' settings and their interaction with food security, and to discuss common needs and opportunities to build more sustainable urban livestock systems in these countries. In total, six papers addressing a variety of relevant Research Topics and presenting cases from Africa and Asia have been published under this Research Topic.

In the first contribution, Lindahl et al. describes the extent and structure of peri-urban smallholder dairy farming in five Indian cities. They point out to significant heterogeneities across the investigated cities in terms of farm size, productivity, and the development and organization of dairy value chains. Likewise, they identify wide variations across the cities with respect to the contribution of dairy production systems to smallholder farmers' income and household food security. These results underscore the crucial role that dairy cooperatives play in developing dairy production systems and enhancing their contribution to improving incomes and food security in tropical countries.

In the second contribution, Pham-Thanh et al. provide an overview of the developments in livestock production in Hanoi city and reviews policies and strategies related to livestock production in the city. They show that small-scale livestock farming systems, which dominate livestock production in Hanoi, are facing a range of challenges that constrain the development of livestock value chains in the city and threaten urban sustainability, including: widespread of animal diseases and epidemic outbreaks, and the lack of consideration of the environmental impact of urban livestock production in urban policies and strategies.

In the third contribution, Mutsami and Karl investigate the impact of commercial rabbit farming on poverty reduction in urban and peri-urban areas in Kenya. They show that commercialization of rabbit farming is significantly associated with a decrease in multidimensional poverty among urban and peri-urban farmers. The study calls for the need for improving the physical infrastructure and enhancing the quality of services provided by credit lending institutions in order to help farmers access affordable credit that enhances their ability to further commercialize their rabbit farming activities and invest in training and capacity building.

In the fourth contribution, Asadu et al. draw on a survey of 210 urban livestock keepers in three states in Southeast Nigeria to assess their knowledge regarding health hazards associated with urban livestock farming. They show that knowledge of these hazards is influenced by extent of farming experience, stock size, and membership in producer organizations, and access to extension services. The study suggests that policy conductive to minimizing the risk of health hazards in livestock farming systems should include elements to raise awareness and knowledge through comprehensive campaigns in urban areas and providing practical training courses adapted to local context. Likewise, there is a need to improve the quality of advisory services to support transforming production to business and profit-oriented livestock enterprise.

In the fifth contribution, Omondi surveys 312 small-scale urban poultry producing households in two medium-sized cities in Kenya, and combines cluster theory and value chain approaches to analyze urban smallholder poultry value chains. He shows small-scale poultry production is a viable and profitable enterprise in urban settings in Kenya, especially among women. The spot market presents the most common form of governance in poultry value chains in the two cities in which trading partners are independent. Formation of producer groups helps smallholder livestock keeper upgrade their activities to adhere to food safety requirements and to lower transaction costs. Finally, the results indicate that extension and veterinary services are crucial to improve the position of producers in the poultry value chain.

In the last contribution, Musundire et al. review national policy frameworks in selected African countries to assess their relevance and stewardship of wild and farmed edible insects as food and feed. Their review focuses on how policies encompassing biodiversity, natural resources, culture, education, research, technology development, trade, health and nutrition could be improved to support inclusivity of edible insects. The study suggests that stewardship of insects could be integrated in national agricultural policies and that regulatory frameworks on the use of edible insects as food and feed are developed. In addition, there is a need for supporting communities to adopt widespread participation in the edible insect value chains and to promote the utilization of indigenous knowledge systems on use of edible insects as food and feed.

In summary, the results of the above-mentioned contributions add to the growing literature on urban livestock systems in LMICs and their interlinkages with food security and urban sustainability. The contributions published in this Research Topic clearly reveal that there are still many aspects to be investigated and understood in relation to needs and opportunities for building sustainability of livestock systems in the context of the LMICs.

Future research aiming to understand how urban livestock systems evolve in the context of rapid urbanization in LMICs is needed and could address the whole continuum of the livestock value chain and the various dimensions that drive food security in these countries and how they possibly interrelate. In light of the increased scope and frequency of disease outbreaks and pandemics, climate and environmental challenges, and geopolitical conflict, which disrupted livestock supply chain activities and threated global food security, we recommend future research to address questions related to the resilience of urban livestock systems in LMICs. This type of research would likely benefit from adopting a more integrated and holistic approaches to account for the complex nature of livestock systems in LMICs while capturing their links with other sectors and systems (such as the health system). Finally, future research could address the preparedness and adaptive capacity of urban livestock systems to potential risks including the constraints and enablers that determine their recovery from such events and enhance their capacity to foster food security and nutrition for the growing populations in LMICs.

## Author Contributions

AA and C-JL wrote the introduction and the conclusion. AA and SB wrote the central part with comments to the cited papers and references. All authors contributed to the editorial and approved the submitted version.

## Funding

This research was supported by a research grant (No. 2016-00350) from the Swedish Research Council for Environment, Agricultural Sciences, and Spatial Planning (FORMAS) and a research grant from AgriFoSe2030.

## Conflict of Interest

The authors declare that the research was conducted in the absence of any commercial or financial relationships that could be construed as a potential conflict of interest.

## Publisher's Note

All claims expressed in this article are solely those of the authors and do not necessarily represent those of their affiliated organizations, or those of the publisher, the editors and the reviewers. Any product that may be evaluated in this article, or claim that may be made by its manufacturer, is not guaranteed or endorsed by the publisher.

## References

[1] Abu Hatab A Cavinato MER Lagerkvist CJ. Urbanization, livestock systems and food security in developing countries: A systematic review of the literature. Food Sec. (2019) 11:279–99. 10.1007/s12571-019-00906-1

[2] Abu Hatab A Ravula P Nedumaran S Lagerkvist CJ. Perceptions of the impacts of urban sprawl among urban and peri-urban dwellers of Hyderabad, India: a Latent class clustering analysis. Environ Dev Sustain. (2021). 10.1007/s10668-021-01964-2

